# Assessment of the psychometric properties of the Italian version of the New Freezing of Gait Questionnaire (NFOG-Q-IT) in people with Parkinson disease: a validity and reliability study

**DOI:** 10.1007/s10072-023-06800-1

**Published:** 2023-04-18

**Authors:** Susanna Mezzarobba, Carola Cosentino, Martina Putzolu, Francescaroberta Panuccio, Giovanni Fabbrini, Donatella Valente, Stefania Costi, Giovanni Galeoto, Elisa Pelosin

**Affiliations:** 1 Department of Neuroscience, Rehabilitation, Ophthalmology, Genetics, Maternal and Child Health, University of Genoa, Genoa, Italy; 2 IRCCS, Ospedale Policlinico San Martino, Genoa, Italy; 3 Department of Human Neurosciences, Sapienza University of Rome, Viale Dell’Università 30, CAP 00185 City Rome, Italy; 4 IRCSS Neuromed, Via Atinense, 18, 86077 Pozzilli, IS Italy; 5 Physical Medicine and Rehabilitation Unit, Azienda Unità Sanitaria Locale - IRCCS Di Reggio Emilia, Via Risorgimento 80, 42123 Reggio Emilia, Italy; 6 Department of Surgery, Medicine, Dentistry and Morphological Sciences, Università Di Modena E Reggio Emilia, 41100 Modena, Italy

**Keywords:** Parkinson disease, Freezing of gait, New Freezing of Gait Questionnaire, Validation, Translation, Italian

## Abstract

**Introduction:**

Freezing of gait (FOG) in Parkinson’s disease (PD) is a challenging clinical symptom to assess, due to its episodic nature. A valid and reliable tool is the New FOG Questionnaire (NFOG-Q) used worldwide to measure FOG symptoms in PD.

**Objective:**

The aim of this study was to translate, to culturally adapt, and to test the psychometric characteristics of the Italian version of the NFOG-Q (NFOG-Q-It).

**Methods:**

The translation and cultural adaptation was based on ISPOR TCA guidelines to finalize the 9-item NFOG-Q-It. Internal consistency was assessed in 181 Italian PD native speakers who experienced FOG using Cronbach’s alpha. Cross-cultural analysis was tested using the Spearman's correlation between the NFOG-Q-It and the Modified Hoehn-Yahr Scale (M-H&Y).

To assess construct validity, correlations among NFOG-Q-It, Movement Disorder Society-Unified Parkinson’s Disease Rating Scale (MDS-UPDRS), Mini-Mental State Examination (MMSE), the Montreal Cognitive Assessment (MoCA), the Falls Efficacy Scale-International (FES-I), the 6-min Walking Test (6MWT), the Mini Balance Evaluation System Test (Mini-BESTest) and the Short Physical Performance Battery (SPPB) were investigated.

**Results:**

The Italian N-FOGQ had high internal consistency (Cronbach’s α = 0.859). Validity analysis showed significant correlations between NFOG-Q-IT total score and M-H&Y scores (*r* = 0.281 *p* < 0.001), MDS-UPDRS (*r* = 0.359 *p* < 0.001), FES-I (*r* = 0.230 *p* = 0.002), Mini BESTest (*r* = -0.256 *p* = 0.001) and 6MWT (*r* = -0.166 *p* = 0.026). No significant correlations were found with SPPB, MOCA and MMSE.

**Conclusion:**

The NFOG-It is a valuable and reliable tool for assessing FOG symptoms, duration and frequency in PD subjects. Results provide the validity of NFOG-Q-It by reproducing and enlarging previous psychometric data.

**Supplementary Information:**

The online version contains supplementary material available at 10.1007/s10072-023-06800-1.

## Introduction

Parkinson Disease (PD) is one of the most common progressive neurodegenerative disorders that affects 0.1% of the general population and 1% of the population over 65 years old, and its prevalence is expected to progressively increase over the coming decades, bringing new social and economic implications on societies [1, 2]. The incidence of the PD increases with age and with an enlarged prevalence in males rather than females [3]. It is characterized by a combination of motor (e.g., bradykinesia, rigidity, tremor and postural instability) and non-motor (e.g., cognitive impairments, sleep disorders, dysautonomia) symptoms. Among motor signs, Freezing of Gait (FOG), defined as the “brief, episodic absence or marked reduction of forward progression of the feet despite the intention to walk” [4] is a disabling symptom that affects more than half of all advanced PD patients [5]. Due to unpredictable and episodic nature, it has a significant impact on fall risk and thus negatively impact on quality of life [6]. The circumstances when FOG occurs are well known (e.g., gait initiation, turning, navigating through a narrow space) and evidence showed that it could be related to environmental triggers, high cognitive demanding conditions and stressful situations [7].

The management of FOG is perceived by clinicians as very challenging. First, the pathogenesis of FOG is not fully understood. Second, to date a broad consensus regarding a possible causal treatment for FOG is still missing, and the best clinical practice include the optimizing dopaminergic therapy, surgical approaches (e.g., Deep brain stimulation) and non-pharmacological therapies (e.g., physiotherapy and exercise training). Third, a gold standard in the assessment of FOG severity and its progression is still missing, even if several behavioral tasks, such as FOG provoking test, or wearable sensors [8] have been used both in clinical and laboratory settings. However, it is important to recall that in clinical routine, high false negative rates of FOG can occur, because patients may attend to walking more consciously [9] and that if results from home-based monitoring are promising, data still reports failure in FOG detection [10]. All together these results call upon the identification of validated measures for FOG.

To date, to evaluate the severity of FOG episodes (e.g., frequency and duration) and to identify the circumstances that trigger this phenomenon (e.g., gait initiation and turning), self-reported questionnaires are the most used tools in the clinical setting [11].

In the past twenty years, the FOG-Q [12] was the most applied questionnaires for assessing FOG. It was validated in several languages, Portuguese (Brazil) [13], Swedish [14], Italian [15] and German [16] and results reported good psychometric properties.

Then, in the 2009, to implement the evaluation of FOG symptoms, an updated version (New Freezing of Gait Questionnaire, NFOG-Q) was proposed by Nieuwboer et al. [6]. In details, one demo video, providing examples of different types of FOG and displaying their duration, together with three additional questions (part III), evaluating FOG impact on activities daily living and QoL, were inserted. Combining the video-clip, describing freezing episodes, with additional questions, investigating how FOG affects patients' daily lives, improved the accuracy of the questionnaire. Indeed, the demo video increase the patients' capacity to recognize and rate FOG significantly and the new questions help clinicians more accurately determine the severity of FOG. To date, published data, reported that NFOG-Q results had a high internal consistency and reliability in people with PD and their caregivers.

Since the NFOG-Q is a valid and widely used questionnaire for assessing FOG, here we translated and evaluated the psychometric properties of the NFOG-Q in an Italian cohort of PD subjects with FOG. The validation of the Italian version of NFOG-Q would assist clinicians in measuring FOG severity more accurately, and it might also support scientific research, by promoting comparisons among published studies [17].

## Methods

The present study was conducted by three research groups from the University of Genova, the Sapienza University of Rome and the Rehabilitation & Outcome Measure Assessment (ROMA) association. This study was divided into two stages. First, the original English version of the NFOG-Q was translated into Italian [18]; second, the translated NFOG-Q was tested for validity and reliability in a cross-sectional study following international guidelines [19].

### Assessment tools

To assess the validity of the Italian version of the NFOG-Q, we looked for correlation between the translated NFOG-Q and clinical outcome measures. The Movement Disorder Society (MDS)-Unified Parkinson’s Disease Rating Scale (UPDRS) is considered the gold-standard in the assessment of PD severity. It is composed by four parts: Part I evaluates the impact of non-motor-disorders on activities of daily living (ADL) with six rater-based items and seven self-assessment questions, Part II evaluates the impact of motor symptoms on daily living with 13 patient-based items, Part III rates the severity of motor symptoms and Part IV assesses motor complications.

The Modified Hoehn-Yahr Scale (M-H&Y) is a new version of the scale developed in 1967 used to describe symptoms progression of PD. It is a five-point scale (from 1 to 5) and include 0.5 increments for points 1 and 2. It starts from stage 1, unilateral involvement, with minimal impairment, up to stage 5, needing a wheelchair or bedridden unless assisted, with severe disability (stage 5). The Mini-Mental State Examination (MMSE) is a widely used screening of cognitive function, characterized by a short duration (about 10 min) and a good reproducibility. It is composed by 11 items divided into 5 sections for testing orientation, attention, memory, language, and visuo-spatial skills. MMSE score is influenced by age and education, for which correction factors are provided. The total score ranges from 0 to 30, where a score of 25 or higher is considered normal.

The Montreal Cognitive Assessment (MoCA) is a tool used to assess general cognitive functions, composed by 30 items in which short-memory, visuo-spatial abilities, executive functions, attention, concentration, working memory, language and orientation to time and place are evaluated. The total maximum score is 30, in which a score of 26 and higher can be considered normal.

The Falls Efficacy Scale-International (FES-I) is a valid instrument for fall risk and fear of falling assessment used at international level. It consists of 16 self-administered items, 10 from the original version (FES) and 6 additional items concerning more demanding physical and social activities. Items are scored on a 4-point scale, with a total score ranging from 16 (no concern about falling) to 64 (severe concern about falling).

The 6-min Walking Test (6MWT) is used to assess the distance walked over 6 min as a submaximal test of endurance. The score is the distance a patient walks in 6 min.

The Mini Balance Evaluation System Test (Mini-BESTest) assesses balance and mobility performance in different conditions (e.g., sit to stand, stand on one leg, change in gait speed etc.). The maximum score is 32, and the higher scores correspond to better balance and functional autonomy. The Short Physical Performance Battery (SPPB) is an assessment tool for evaluation of lower extremity functioning and balance ability in older population. It consists of 3 sections: (1) Balance; (2) Gait and (3) Sit to stand assessments. The total score ranges from 0 to 12, in which higher scores represent better lower extremity function and balance.

### Translation and cultural adaptation

The NFOG-Q is an updated version of the FOG-Q and it is used to assess specific clinical aspects of freezing (i.e., frequency, duration, and impact on quality of life). The NFOG-Q is composed by one video, showing examples of different types and duration of freezing episodes, and 9 questions. It is divided into three parts: Part I, “Distinction Freezer – non-Freezer” (item 1), which classifies subjects as freezers (FR) or non-freezers (NFR), Part II, “Freezing severity” (items 2–6), which assess FOG severity in terms of frequency and duration of FOG episode during gait initiation and turning and Part III “Freezing impact on daily life” (items 7–9), which assess the impact of walking independence and fear of falling. Total score ranges from a 0 to 28 points, where 0 indicate the absence of FOG and the highest the score the greatest is FOG severity.

After having received the permission of the developers of the original version of the NFOG-Q, the questionnaire was adapted into Italian (NFOG-QIt) following the “Translation and Cultural Adaptation of Patient Reported Outcomes Measures-Principles of Good Practice” guidelines [17]. The translation process included three steps: (1) two native English translators who were familiar with the examined topics have independently translated the questionnaire into Italian (forward translation); (2) one native Italian translator, aware of the subject matter, chose the best translation in order to create the final Italian version of the tool; and (3) a bilingual person with a certificated knowledge of the English language translated the text back into English (backward translation). The forward and backward translations were reviewed by qualified Italian and English-speaking clinicians before agreement on the final version. Final step consisted of having the approval of the Italian version by the main authors of the NFOG-Q.

### Participants

The sample was recruited from the Department of Neuroscience (DINOGMI) of Genova University. The following inclusion criteria were applied to the eligible and interested persons: (i) diagnosis of idiopathic PD (according to the United Kingdom Parkinson’s Disease Society Brain Bank criteria); (ii) age > 50 but < 90 years; the presence of FOG, evaluated with the FOG-Q, (iii) Modified-Hoehn &Yahr stages of almost 2.0; (iv) no severe cognitive impairment (MMSE score > 22); (v) have the ability to communicate and understand Italian language. Exclusion criteria were no other neurological and psychiatric disturbances or orthopedic conditions that severely restricts walking. According to the ethical principles of the Declaration of Helsinki, participants interested in taking part in the study, were informed about the study’s purpose and methods, and their interest was recorded. All participants provided their written informed consent prior to inclusion.

### Testing procedure

Patients were asked to self-complete the NFOG-QIt (no help from caregivers or assessors). After having completed the questionnaire, motor and cognitive performances (MDS-UPDRS, M-H&Y, MMSE, MoCA, FES-I, 6-Mwt, SPPB) were measured by clinicians and researchers (all expert in movement disorders) involved in the study. All the evaluations were performed during the ON STATE (about 45–60 min after the LDOPA dose).

### Data analyses

All statistical analyses were performed with SPSS 23.0. P-values < 0.05 were considered as threshold for statistical significance. Data collected were reported as frequency tables, means, and standard deviation (SD). The psychometric characteristics of the tool were assessed based on the Consensus-Based Standards for the Selection of Health Status Measurement Instrument (COSMIN) checklist [18]. The internal consistency was examined by Cronbach’s alpha (α) in order to evaluate the interrelatedness of the items and the homogeneity of the scale. Alphas higher than 0.70 score were considered acceptable as an indicator of the satisfactory homogeneity of each item within the total scale.

To perform the cross-cultural analysis non-parametric correlations between the total and the second and third subscales score of the NFOG-QIt and the M-H&Y scores (ranked as 2, 2.5 and 3) was run, assuming that a worsening of the disease may be correlated to a greater manifestation and incidence of FOG episodes.

Construct validity was calculated using the Pearson correlation coefficient (r) computed among NFOG-Q-IT and the clinical scales (MDS-UPDRS, M-H&Y, MMSE, MoCA, FES-I, 6-Mwt, SPPB) separately. The following ranges were used to interpret the results: 0 indicates no linear relationship; + 1/ − 1 indicates a perfect linear positive/negative relationship; values between 0 and 0.3 (0 and − 0.3) indicate a weak linear positive (negative) relationship through a shaky linear rule; values ranging from 0.3 to 0.7 (− 0.3 and − 0.7) indicate a moderate positive (negative) linear relationship through a fuzzy-firm linear rule; and values between 0.7 and 1.0 (− 0.7 and − 1.0) indicate a strongly positive (negative) linear relationship through a firm linear rule.

## Results

To adapt the translated version to the Italian culture, the translation was adjusted, and items were modified to minimize differences from the original version, when needed. Minor changes (slight differences in questions’ phrasing) were discussed among clinician expert in movement disorders and the translators. Then the final Italian version was approved.

One hundred and eighty-one individuals with PD and FOG (Males:112; Age, mean ± SD: 70.5 ± 3.54 years; Disease duration: mean ± SD: 9.97 ± 6.55 years) recruited over a period of 12 months between 2020 e 2021, participated in this study. Mean of total score of NFOG-Q was 15.03 (± 5.84) and ranged between 3–28. The participants H&Y scores ranged from 2 to 3 and no individuals were in 1 and 4 H&Y stages. Demographic and clinical characteristics of participants are reported in Table 1.

**Table 1:** Demographical and clinical characteristics of participants (*n* = 181)

Outcome Measure	Mean		SD
NFOG-Q (score)	15.03	±	5.84
Age (yrs)	70.5	±	3.54
Gender (M /F)	112 / 69		
Disease duration (yrs)	9.97	±	6.55
H&Y stage (2;2,5;3) (score)	2.54	±	0.45
MDS-UPDRS Part I (score)	12.48	±	5.82
MDS-UPDRS Part II (score)	18.75	±	7.20
MDS-UPDRS Part III (score)	31.52	±	12.91
MDS-UPDRS Part IV (score)	4.08	±	4.18
MDS-UPDRS Tot. (score)	66.85	±	21.75
MMSE (score)	28.22	±	1.66
MoCA (score)	24.08	±	3.59
FES-1 (score)	36.22	±	11.74
MiniBestest (score)	21.22	±	5.63
6MWT (m)	116.14	±	33.13
SPPB (score)	8.65	±	2.42
H&Y	Frequency		
stage 2	66		
stage 2,5	30		
stage 3	85		

*SD* standard deviation, *NFOG-Q* New Freezing of Gait Questionnaire, *yrs* years, *H&Y* Hoehn and Yahr Scale, *UPDRS* Unified Parkinson Disease Rating Scale, *MMSE* Mini Mental State Examination, *MoCA* Montreal Cognitive Assessment, *FES-1* Fall Efficacy Scale, *6MWT* Six Minute Walking Test, *SPPB* Short Physical Performance Battery

**Fig. 1:**
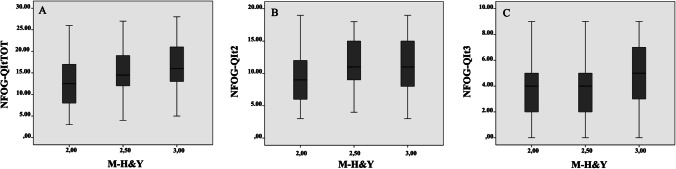
Cross-cultural validity analysis. Spearman's rank correlation coefficient between the Italian version of New Freezing of gait Questionnaire (NFOG-QIt) total score (**A**), NFOG-QIt Part 2 score (**B**), NFOG-QIt Part 3 score (**C**) and the Modified Hoehn-Yahr Scale (M-H&Y)

The NFOG-QIt had a good reliability, with a Cronbach’s α of 0.859. Internal consistency was calculated for the total score and for each sub-item scores. Results of Cronbach’s α are reported in Table 2. Regarding cross-cultural validity, results for NFOG-QIt total score showed a significant correlation across the M-H&Y scores (*r* = 0.281 *p* < 0.001). Similarly, an increment was found when the second (i.e., Freezing severity: rs = 0.220 *p* = 0.003) and third (i.e., Freezing impact on daily life: *r* = 0.299 *p* < 0.001). Results are shown in Fig. 1.

**Table 2:** Internal Consistency of the NFOG-QIt standardized Cronbach’s α value for each ordinal-scale item

Question	Mean	Cronbach's alpha
2. How frequently do you experience freezing episodes?	12.74	0.844
3. How frequently do you experience freezing episodes during turning?	12.48	0.841
4. How long is your longest freezing episode during turning?	13.32	0.826
5. How frequently do you experience episodes of freezing when initiating the first step?	12.46	0.848
6. How long is your longest freezing episode when initiating the first step?	13.28	0.850
7.How disturbing are the freezing episodes for your daily walking?	13.13	0.843
8. Do the freezing episodes cause feelings of insecurity and fear of falling?	13.51	0.841
9. Are your freezing episodes affecting your daily activities?	13.93	0.841

**Fig. 2:**
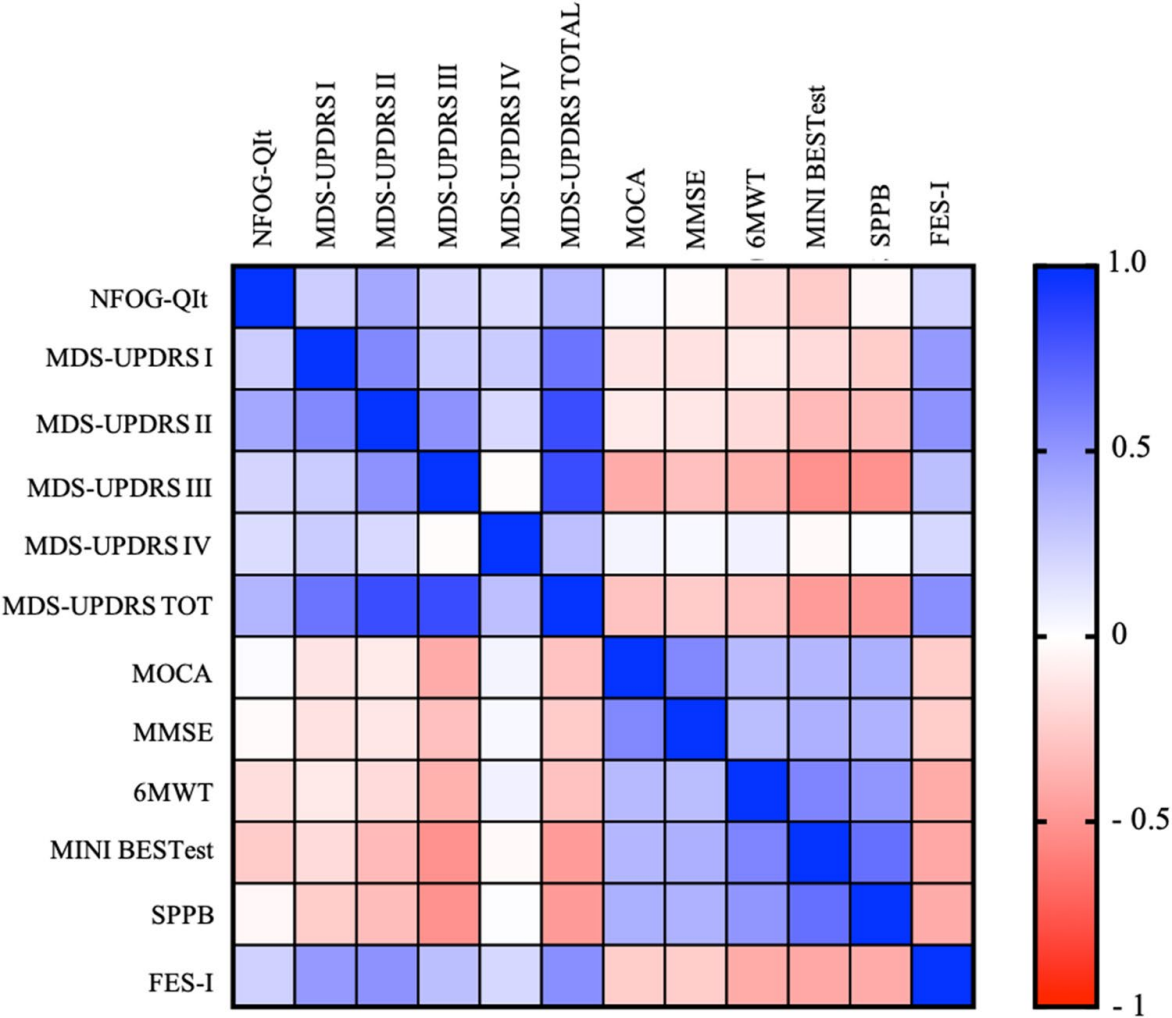
Heatmap representation of correlation coefficients between the NFOG-QIt total score (and items) and clinical scales. Correlation coefficient (Pearson r) are color-coded as shown in the vertical bar. Movement Disorders Society-Unified Parkinson’s Disease Rating Scale (MDS-UPDRS), Montreal Cognitive Assessment (MoCA); Mini Mental State Examination (MMSE); 6-minuteWalking Test (6MWT); Mini Balance Evaluation System Test (Mini-BESTest); SPPB (Short Physical Performance Battery) and Falls Efficacy Scale-International (FES-I). Indexes on the bar: 0 indicates no linear relationship; + 1/ − 1 indicates a perfect linear positive/negative relationship.

The construct validity analysis showed a significant correlation between the total scores of the NFOG-QIt and the MDS-UPDRS (*r* = 0.359 *p* < 0.001). When testing the relationship between the NFOG-QIt and the MDS-UPDRS sub-scores several significant correlations were found: the strongest was found with MDS-UPDRS part II (*r* = 0.421 *p* < 0.001) and the weakest with MDS-UPDRS part IV (*r* = 0.171 *p* = 0.021). In addition, the total NFOG-QIt score was correlated with FES-1 (*r* = 0.230, *p* = 0.002) and Mini-BESTest (*r* = -0.256, *p* = 0.001), 6MWT (*r* = -0.166, *p* = 0.026) total scores. No significant relationship was found between the NFOG-QIt and the SPPB total score (*r* = -0.43 *p* = 0.569). Finally, in line with previous results, no significant correlations were found between NFOG-QIt and MOCA and MMSE total scores. Results of the construct validity are depicted in Fig. 2. Details of correlation results are reported in Supplementary Materials (Table S1).

## Discussion

The aim of this study was to translate, to cross cultural adapt, and to test the psychometric properties the Italian version of the NFOG-Q. Overall, results showed that the NFOG-QIt is a reliable and valid tool to assess freezing of gait severity and its impact on daily life activities in individuals with PD. The NFOG-Q-It presented a high internal consistency (α = 0.859) that was in line with previous results of the original (English: range between 0.89 and 0.9) [6] and later versions (Italian α = 0.95 German α = 0.83) of FOG-Q [15, 16]. Also, our results were comparable with those reported by the English (α = 0.84) [6] and the German (α = 0.84) NFOG-Q version [11].

In line with the NFOGQ German validation, the cross-cultural analysis (Fig. 1) showed significant correlation between the NFOG-QIt total and the M-H&Y scores, revealing that the PD progression is associated with a worsening of freezing symptoms. This result was confirmed when the M-H&Y scale was correlated with part II (“Freezing severity”) and part III (“Freezing impact on daily life”) sub scores. These results are supported by previous studies showing an increased FOG prevalence according to the stage of the disease [20] and are in line with those reported in the validation of FOG-Q in other languages (Italian, Swedish) [14, 15].

The validity of the NFOG-QIt was supported by high positive correlations with the MDS-UPDRS total and sub-total scores (Part I to Part IV, Fig. 2). More severe the PD symptoms are, worst FOG severity is. These results are consistent with previous studies showed that several motor (e.g., rigidity and bradykinesia) and non-motor (e.g., sleep disorders cognitive or emotional status) features of PD correlate with frequency and FOG severity [21]. Specifically, we found a strong correlation between NFOG-QIt and Part I (i.e., “Non motor aspects of daily living”) showing as PD patients reporting more severe non-motor impairments (e.g., cognition, anxiety, pain, fatigue, orthostatic hypotension) had a higher FOG severity. These results underline how non-motor symptoms could be more severe in PD with FOG and are supported by recent evidence suggesting that cognitive symptoms and emotional status may contribute to worse FOG events [22–25]. Regarding cognitive aspect, a recent review [24, 25] showed that PD subjects with FOG manifest worse global cognition, in particular executive functions, visuospatial ability, and memory than PD subjects without FOG. However, in line with previous results, it should be noted that here neither MoCA nor MMSE scores correlated with NFOG-QIt score.

Regarding the emotional status it has been reported that emotions, anxiety and fear primarily, might trigger FOG episodes and exacerbate its symptoms (e.g., increase its duration). Taking together these results support the existence of a relationship among FOG, cognitive deficits, however, the direction of the causality and the underlying pathophysiological mechanisms are still open questions [22–26]. A significant correlation was also found with MDS-UPDRS Part II (i.e., “Motor Aspects of Experiences of Daily Living”) data and NFOG-QIt scores. PD patients who referred a greater disability in daily living due to motor symptoms where those with higher FOG symptoms. This finding is in line with recent data reporting a strong association of FOG with severe functional dependency in the activity of daily living [26].

The observation of a strong relationship between NFOG-QIt and motor symptoms, measured with Part III subs-core, is not surprising. Indeed, it is well established that the incidence and the number of FOG episodes correlate with Postural Instability Gait Disorders (PIGD) related symptoms, such as bradykinesia and rigidity, and with gait impairments and falls [7, 21].

Finally, when a possible relationship between FOG and motor complications (MDS-UPDRS part IV) was investigated, a significant positive correlation was found. As reported in a recent review, [20] the “OFF-state” FOG is frequently seen in the stage of early-fluctuations, the “OFF–ON” FOG usually appears with disease progression whereas the so called “Biphasic” FOG [27] described as Levo-Dopa-induced FOG, emerges in the OFF and ON transition phase. Therefore, it could be assumed that FOG symptoms may be more severe with the emergence of motor fluctuation, and with longer OFF state periods. Regarding a possible relationship between dyskinesias and FOG severity, results from previous studies are still inconclusive. In a recent study, Aktürk and coworkers [7] found a significant relationship between FOG and motor fluctuations, but when a correlation between dyskinesia and FOG severity was tested, no significant results emerged. Conversely, the so called “Supra-ON freezing of gate” [28] seems to emerge more frequently in PD patients with diphasic dyskinesia. Despite that, due to the complexity of both dyskinesias and FOG phenomenology, our results may not support neither one nor the other hypothesis.

When potential relationships between clinical scales related to balance, gait, mobility, and fear of falling and NFOG-QIt were investigated, significant correlations emerged. Indeed, in line with previous finding [11] we found that PD patients with a higher FES-I score had a higher NFOG-It score and that poor postural control (e.g., lowest Mini-Best score) and gait difficulties (e.g., shortest distance in the 6WT) were associated with more severe FOG. These results are consistent with a large amount of evidence reporting a higher falls number in PD with FOG compared to those without FOG [29] as well as with evidence reporting postural control and balance deficit [30] in PD with FOG. Finally, although in the NFOG Chinese validation [31] a significant correlation between the FOG and general physical performance (SPPB) was found, our statistical analysis did not reach statistical significance.

### Limitations of the study

Certain limitations of our study are to be acknowledged. First, in our participants with severe cognitive impairments were not included. However, NFOG-Q is a self-reported questionnaire that required patients to recall FOG episodes and to evaluate its severity over the past month, therefore severe cognitive impairments might skew the results. Despite that, to evaluate FOG impact on daily living in PD with major cognitive deficit, it might be useful to administer NFOG-QIt to their caregivers. Second, due to SARS-CoV-2 pandemic, test–retest reliability was not performed. Future studies should include a test–retest analysis to evaluate the reliability of the NFOG-QIt in individuals with PD. Third, has already reported in the German translation [11], NFOG-Q is a self-report instrument, therefore it is influenced by patient’s perception of symptoms. However, it should be considered that self-report instruments are needed in the clinical setting because can assist a clinician to better characterize patients’ symptoms or to evaluate the impact on their daily living activities and that so far, a gold-standard measure that can objectively evaluate FOG episodes is still missing [6]. Finally, correlation analyses with outcome measures related to medication (e.g., LEDD), other PD symptoms (e.g., dysautonomia) and the presence of comorbidities were not performed, since data were not collected systematically. Further studies, exploring possible relationship between N-FOG scores and other clinical characteristics of PD subjects would be helpful to better contextualize the results obtained.

## Conclusion

To date, the NFOG-Q is the most used clinical tools to assess FOG severity and its impact to activities of daily living in PD. Our results proved that NFOG-QIt is a reliable instrument for the assessment of FOG severity and extended previous results adding some piece of information about the relationship between FOG and clinical and functional scales. Having a validated and reliable measurements is essential both for clinical and research activities also for planning a better and more tailored treatment based on patients’ needs.

## Supplementary Information

Below is the link to the electronic supplementary material.

Supplementary file1 (DOCX 16 KB)
