# Supplementary material for: Assessment of the psychometric properties of the Italian version of the New Freezing of Gait Questionnaire (NFOG-Q-IT) in people with Parkinson disease: a validity and reliability study
Source: Neurol Sci. 2023 Apr 18;44(9):3133–40. doi: 10.1007/s10072-023-06800-1 (PMC10112304; doi:10.1007/s10072-023-06800-1)
Supplement: Supplementary file 1 — Supplementary file1 (DOCX 16 KB) [file 10072_2023_6800_MOESM1_ESM.docx]

**Table S1: Correlation analyses results.** Correlation coefficients between the NFOG-QIt total score (and items) and clinical scales.

| **Clinical Scale** | **Pearson (r)** | **p value** |
| --- | --- | --- |
| NFOG-QIt |  |  |
| MDS-UPDRSI | 0.244 | 0,001 |
| MDS-UPDRSII | 0.421 | 0,000 |
| MDS-UPDRSIII | 0.205 | 0,205 |
| MDS-UPDRSIV | 0.171 | 0,171 |
| MDS-UPDRS TOTAL | 0.359 | 0,000 |
| MOCA | 0.017 | n.s |
| MMSE | -0.027 | n.s |
| 6MWT | -0,166 | 0,026 |
| MINIBESTest | -0.256 | 0,001 |
| SPPB | -0.043 | n.s |
| FES-I | 0.230 | 0,002 |
| Movement Disorders Society-Unified Parkinson’s Disease Rating Scale (MDS-UPDRS), Montreal Cognitive Assessment (MoCA); Mini Mental State Examination (MMSE); 6-minuteWalking Test (6MWT); Mini Balance Evaluation System Test (Mini-BESTest); SPPB (Short Physical Performance Battery) and Falls Efficacy Scale-International (FES-I). ​ | | |
